# *CD34^hi^* subset of synovial fibroblasts contributes to fibrotic phenotype of human knee osteoarthritis

**DOI:** 10.1172/jci.insight.183690

**Published:** 2025-01-23

**Authors:** Junya Miyahara, Yasunori Omata, Ryota Chijimatsu, Hiroyuki Okada, Hisatoshi Ishikura, Junya Higuchi, Naohiro Tachibana, Kosei Nagata, Shoichiro Tani, Kenichi Kono, Kohei Kawaguchi, Ryota Yamagami, Hiroshi Inui, Shuji Taketomi, Yasuhide Iwanaga, Asuka Terashima, Fumiko Yano, Masahide Seki, Yutaka Suzuki, Roland Baron, Sakae Tanaka, Taku Saito

**Affiliations:** 1 Sensory & Motor System Medicine,; 2 Bone and Cartilage Regenerative Medicine,; 3 Center for Disease Biology and Integrative Medicine, and; 4 Department of Chemistry and Biotechnology, Graduate School of Engineering, The University of Tokyo, Tokyo, Japan.; 5 Laboratory of Systems Genomics, Department of Computational Biology and Medical Sciences, The University of Tokyo, Kashiwa, Japan.; 6 Department of Medicine, Harvard Medical School and Endocrine Unit, MGH, Boston, Massachusetts, USA.

**Keywords:** Bone biology, Inflammation, Osteoarthritis

## Abstract

Osteoarthritis (OA) shows various clinical manifestations depending on the status of its joint components. We aimed to identify the synovial cell subsets responsible for OA pathophysiology by comprehensive analyses of human synovium samples in single-cell resolution. Two distinct OA synovial tissue groups were classified by gene expression profiles in RNA-Seq: inflammatory and fibrotic. The inflammatory group exhibited high expression of inflammatory cytokines, histologically inflammatory infiltrate, and a more severe pain score. The fibrotic group showed higher expression of fibroblast growth factor (FGFs) and bone morphogenetic proteins (BMPs), showed histologically perivascular fibrosis, and showed a lower pain score. In single-cell RNA-Seq (scRNA-Seq) of synovial cells, *MERTK^lo^CD206^lo^* macrophages and *CD34^hi^* fibroblasts were associated with the inflammatory and fibrotic groups, respectively. Among the 3 fibroblast subsets, *CD34^lo^THY1^lo^* and *CD34^lo^THY1^hi^* fibroblasts were influenced by synovial immune cells, whereas *CD34^hi^* fibroblasts were influenced by mural and endothelial cells. Particularly, in *CD34^hi^* fibroblast subsets, *CD34^hi^CD70^hi^* fibroblasts promoted proliferation of Tregs, potentially suppressing synovitis and protecting articular cartilage. Elucidation of the mechanisms underlying the regulation of these synovial cell subsets may lead to novel strategies for OA therapeutics.

## Abstract 2

Synovial cell subsets associated with osteoarthritis phenotypes were identified through single-cell RNA sequencing of human synovium samples, distinguishing inflammatory and fibrotic groups.

## Graphical abstract

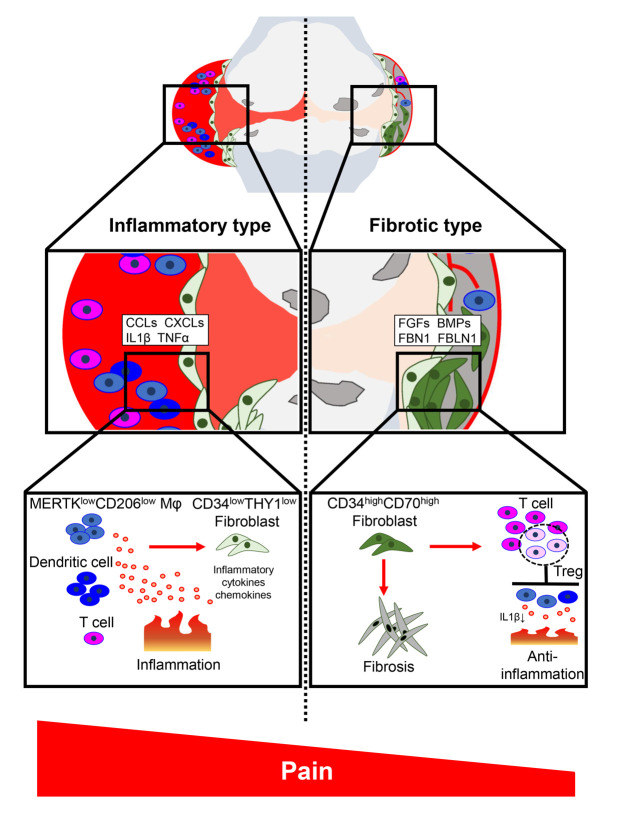

## Introduction

Osteoarthritis (OA), the most prevalent joint disorder, is characterized by the degeneration of articular cartilage. Joint pain that restricts daily activities is the most common symptom of OA. Many epidemiological studies show that severe radiographic OA is associated with increased joint pain (1); however, it is noteworthy that more than half of people with severe OA reported not having pain on most days in the previous month (2). Pain does not worsen inexorably and may not follow a pattern (1). Increasingly, evidence is showing that articular structures other than cartilage, such as synovium and subchondral bone, also play essential roles in the pathophysiology and symptomology of OA (3, 4). For example, synovitis determined by MRI influences the progression of knee OA (5, 6) and correlates with pain (7). A series of mechanistic studies indicated that catabolic enzymes and proinflammatory molecules released from inflamed synovium are associated with articular cartilage degeneration and joint pain (3, 4).

Comprehensive analyses of human synovial tissues using bulk RNA-Seq and single-cell RNA-Seq (scRNA-Seq) have displayed an association between synovial fibroblasts and macrophages in the pathogenesis of rheumatoid arthritis (RA) (8, 9). Mizoguchi et al. reported 3 fibroblast subsets: CD34^–^THY1^–^, CD34^–^THY1^+^, and CD34^+^. Among them, CD34^–^THY1^+^ fibroblasts are abundant in human RA synovium and have a proinflammatory and invasive phenotype (8). Alivernini et al. revealed 2 synovial tissue macrophage subpopulations in human RA synovium: proinflammatory MERTK^–^CD206^–^ and antiinflammatory MERTK^+^CD206^+^ macrophages (9). MERTK^–^CD206^–^ macrophages are related to flare, while MERTK^+^CD206^+^ macrophages are associated with remission maintenance (9). In OA research, Tang et al. identified DPP4^+^ mesenchymal cells as a common progenitor for synovial lining fibroblasts through scRNA-Seq of human OA synovial samples and reported that Apolipoprotein E (APOE) signaling from intermediate fibroblasts and macrophages was identified as a critical regulatory factor (10). Nevertheless, the roles of synovial cells in OA pathophysiology have not yet been understood, compared with recent progress in RA. We hypothesize that the types of cells present in the synovium of OA knees are diverse and that these differences in cell types may be related to phenotypes and symptoms.

Here we performed RNA-Seq using synovial tissues obtained from patients with OA undergoing arthroplasty and classified them according to gene expression patterns. We examined associations of the OA subgroups with their clinical manifestations. To investigate synovial cell profiles, we performed scRNA-Seq using 4 synovial tissues, including these subgroups of gene expression patterns, and analyzed alterations in fibroblast and immune cell subsets. We estimated interactions between the synovial cell subsets as well as between synovial cells and articular chondrocytes by merging deposited scRNA-Seq datasets. Finally, we analyzed the function of distinct synovial fibroblasts closely related to the resolution of knee OA synovitis.

## Results

### Knee OA synovium tissues are classified into 2 groups.

**Figure 1:**
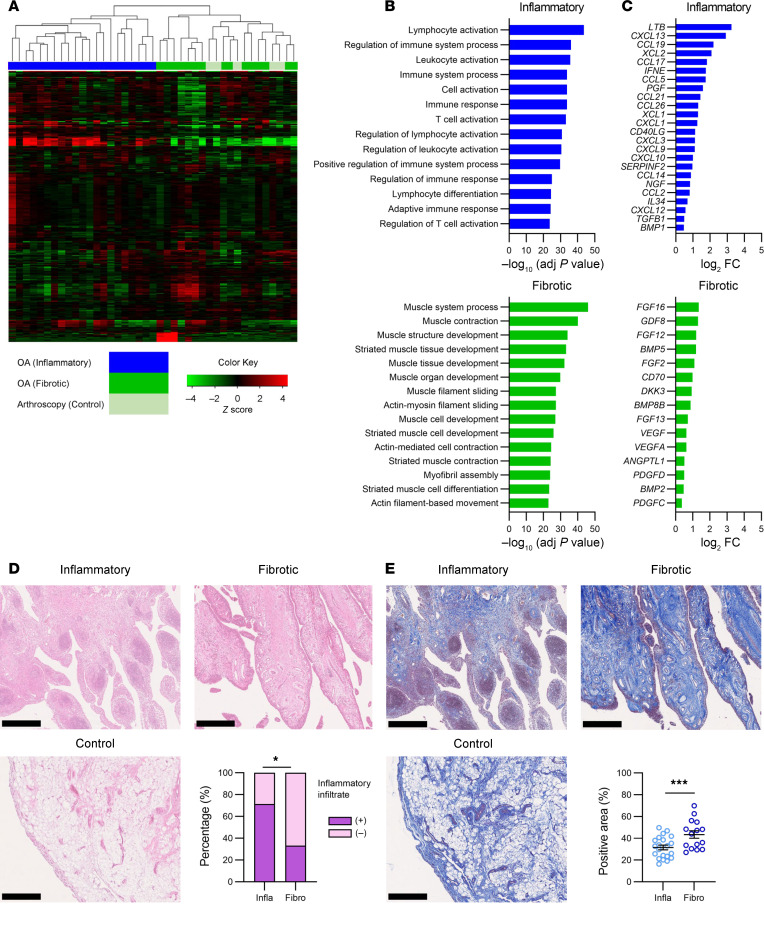
Classification of human knee OA synovium by RNA-Seq and histological evaluation. (**A**) Hierarchical clustering of 41 synovium samples from 36 TKA and 5 arthroscopy patients. (**B**) GO analysis of the 2 groups of OA synovium. (**C**) DEGs of the 2 groups of OA synovium, classified as cytokines, chemokines, and growth factors. (**D**) H&E staining of synovial tissues. Representative images are shown. The right lower panel indicates the percentage of specimens with inflammatory infiltrates in the 2 types of OA synovium. Infla, inflammatory type; Fibro, fibrotic type. Infla, *n* = 21; Fibro, *n* = 15. Fisher’s exact test. *P < 0.05. Scale bar: 100 μm. (**E**) Masson’s trichrome staining of synovial tissues. Representative images are shown. The right lower panel indicates the percentage of positive areas in the 2 types of OA synovium. Infla, *n* = 21; Fibro, *n* = 15. Mann-Whitney *U* test (***P < 0.001). All data were expressed as dot plots and mean ± SEM. Scale bar: 100 μm.

We performed RNA-Seq using synovial tissues obtained from 36 patients with knee OA undergoing primary total knee arthroplasty (TKA) and 5 patients undergoing arthroscopy 1 year after anterior cruciate ligament reconstruction. According to their expression profiles, all 41 synovial tissues were divided into 2 groups (Figure 1A and Supplemental Figure 1, A and B; supplemental material available online with this article; https://doi.org/10.1172/jci.insight.183690DS1). We then extracted differentially expressed genes (DEGs) from the 2 groups and compared their characters. Gene ontology (GO) analysis showed that inflammation- and immune response–related terms were elevated in one group of expression profile, while terms relating to myofibers and muscles were elevated in the other group (Figure 1B). Accordingly, DEGs in one group included cytokines and chemokines related to inflammation, and DEGs in the other type included fibroblast growth factor (FGFs) and bone morphogenetic proteins (BMPs) (Figure 1C and Supplemental Figure 1C). Therefore, we named the former expression profile type as “inflammatory” and the latter as “fibrotic.” Notably, all synovial samples from arthroscopy patients were classified to the fibrotic group (Figure 1A).

H&E staining of the synovial tissues showed that inflammatory infiltration was more frequently observed in the inflammatory type (Figure 1D). In contrast, collagen fibers determined by Masson’s trichrome staining was more widely detected in the fibrotic type of synovial tissues (Figure 1E). Overall, these data indicate 2 types of expression profile in knee OA synovium: inflammatory and fibrotic.

### Pain score is worse in the patients from the inflammatory group.

**Figure 2:**
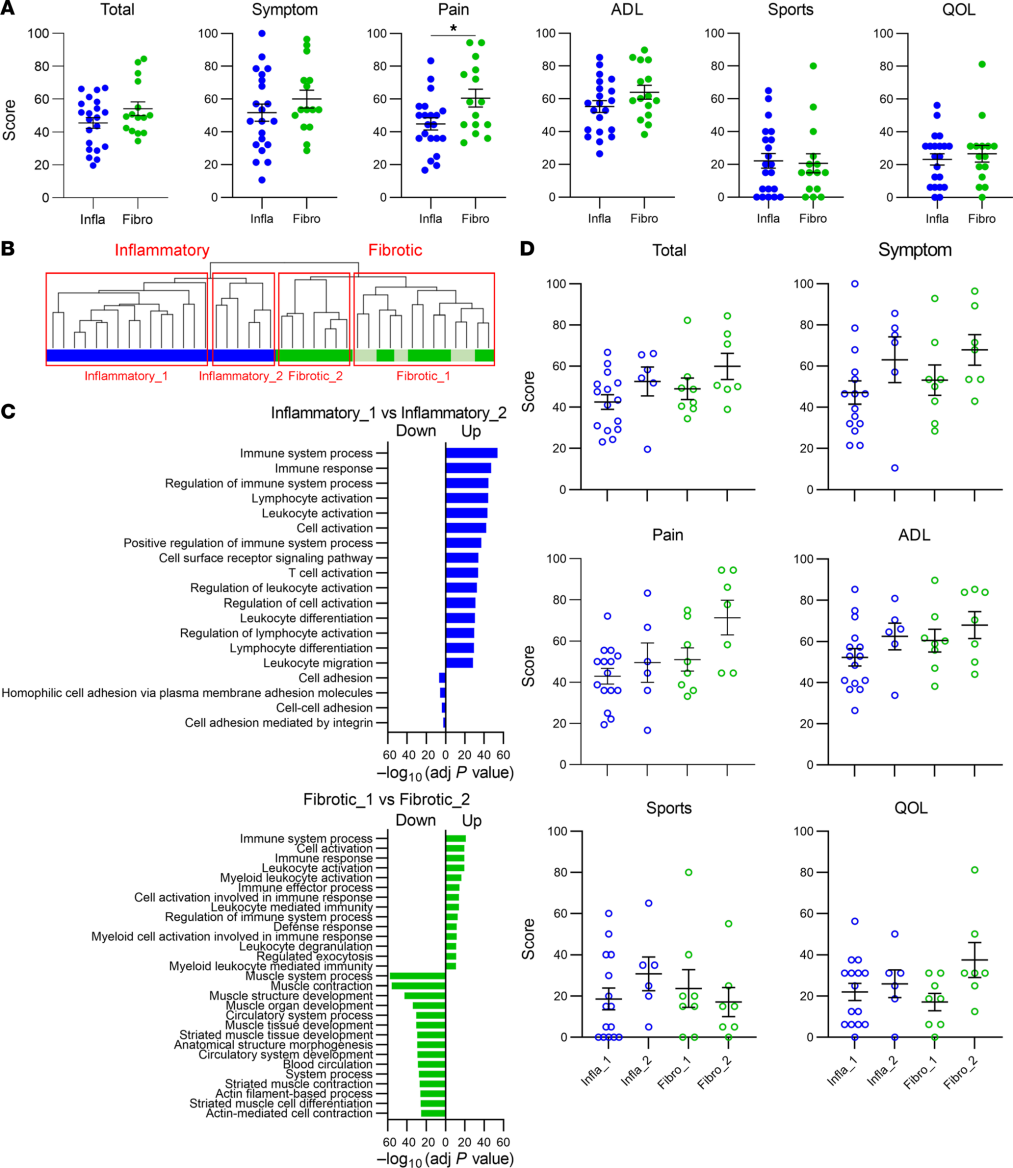
Comparison of clinical scores between patients with knee OA with different types of synovium. (**A**) Comparison of KOOS total scores and 6 subscale scores, including Symptom, Pain, ADL, Sports, and QOL, between patients with inflammatory and fibrotic groups of synovium. Infla, inflammatory group; Fibro, fibrotic group. Infla, *n* = 21; Fibro, *n* = 15. Mann-Whitney *U* test (*P < 0.05). (**B**) Subclustering of the 2 synovium types. (**C**) GO analysis of the 2 synovium subtypes within the inflammatory or fibrotic types. (**D**) KOOS between patients with 4 subtypes of OA synovium. Infla_1, *n* = 15; Infla_2, *n* = 6; Fibro_1, *n* = 8; Fibro_2, *n* = 7. Mann-Whitney *U* test. All data were expressed as dot plots and mean ± SEM.

We compared clinical characteristics and knee clinical scores between patients with the 2 groups of synovial tissues. There were no significant differences between the groups regarding age at surgery, sex, BMI, and Kellgren-Lawrence (KL) grade determined by plain knee radiographs (Supplemental Table 1). According to the knee injury and OA outcome score (KOOS) subscale, pain was significantly worse in the inflammatory group (Figure 2A). The KOOS total score and the Symptom and Activities of Daily Living (ADL) subscales were not significantly different (Figure 2A). To illustrate the trends in relationship between gene expression profiles and KOOS subscales, we further clustered the samples into 4 groups based on hierarchical clustering results in RNA-Seq and compared their GO terms (Figure 2B, Supplemental Table 2, and Supplemental Figure 2). The inflammatory groups were divided into 2 subgroups; GO terms related to immune response and immune activities markedly elevated in the inflammatory_1 (Infla_1) subgroup, while the Infla_2 was characterized by GO terms related to cell adhesion (Figure 2C). Similarly, GO terms related to muscle contraction were predominantly enriched in the fibrotic_2 (Fibro_2) subgroup, while immune-related terms relatively elevated in the Fibro_1 subgroup (Figure 2C). Although there were no statistically significant differences, the mean scores of all KOOS subscales (except Sports) were higher in the Fibro_2 group, while the other subscales were lower in the inflammatory_1 group (except quality of life [QOL] and Sports; Figure 2D), implying that patients in the Fibro_2 and inflammatory_1 group tended to have better and worse clinical symptoms, respectively. These data suggest that genes related to immune response, immune cell activation, muscle, and myofiber in the synovium may be associated with clinical symptoms of knee OA.

### scRNA-Seq of synovial cells identify characteristic clusters for OA subgroups.

**Figure 3:**
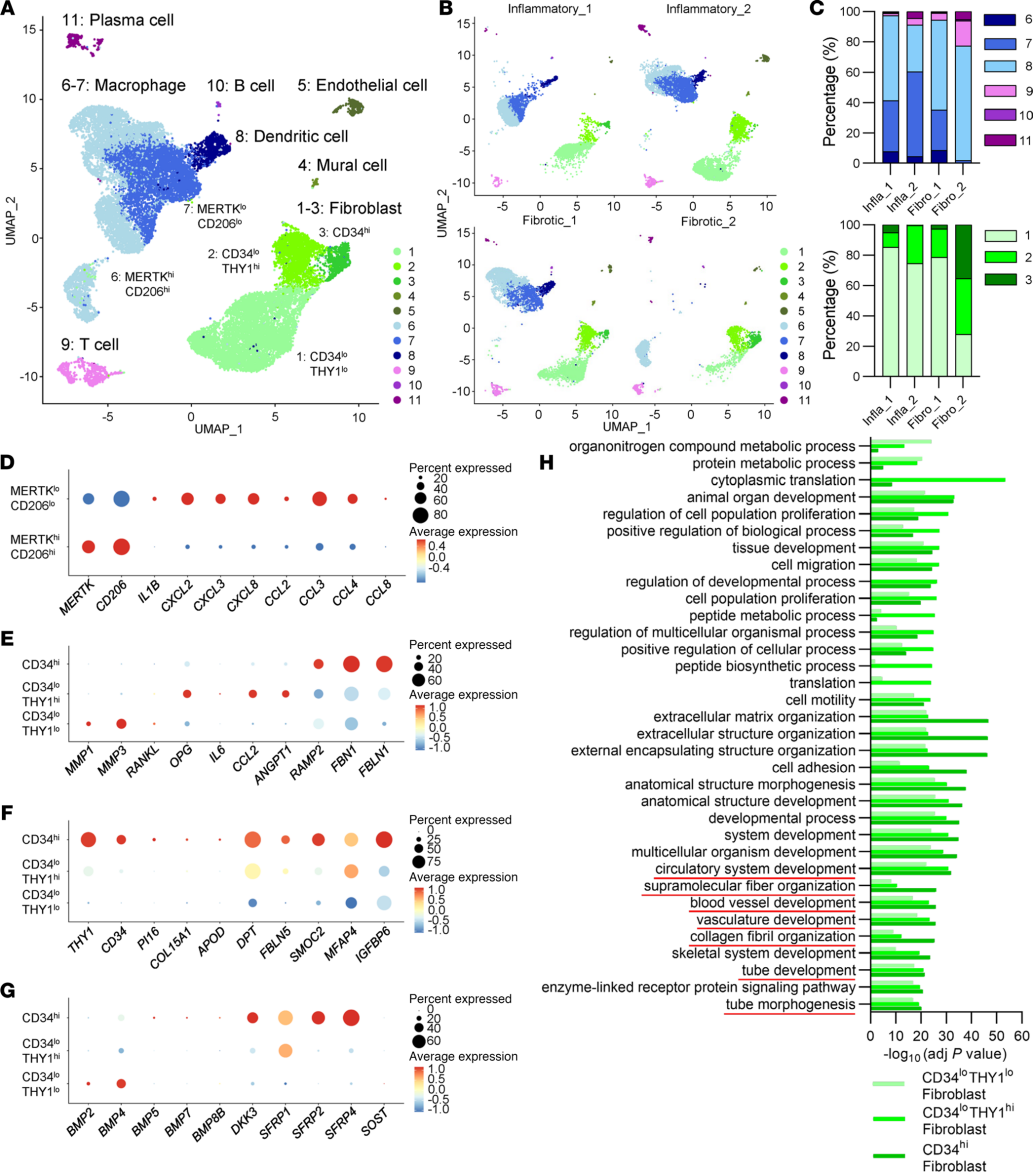
Single cell–based transcriptional profiling of synovial cell subsets. (**A**) UMAP of 11 synovial cell clusters identified by scRNA-Seq. (**B**) UMAP of 11 synovial cell clusters, split by each sample. (**C**) Percentage of immune cell and fibroblast subsets in the 4 synovium samples. Infla, inflammatory group; Fibro, fibrotic group. (**D**) Dot plots of representative cytokine and chemokine genes in *MERTK^lo^CD206^lo^* and *MERTK^hi^*CD206*^hi^* macrophages. (**E**) Dot plots of representative genes in *CD34^lo^THY1^lo^*, *CD34^lo^THY1^hi^*, and *CD34^hi^* fibroblasts. (**F**) Dot plots of stable state marker genes in *CD34^lo^THY1^lo^*, *CD34^lo^THY1^hi^*, and *CD34^hi^* fibroblasts. (**G**) Dot plots of BMPs and WNT inhibitor genes in *CD34^lo^THY1^lo^*, *CD34^lo^THY1^hi^*, and *CD34^hi^* fibroblasts. (**H**) GO analysis of 3 fibroblast subsets.

We selected 1 representative patient from each subgroup and performed scRNA-Seq using 4 synovial samples (Supplemental Table 3 and Supplemental Figure 3). Uniform manifold approximation and projection (UMAP) plotting identified 11 distinct cell clusters, which were characterized based on their expression profiles (Figure 3A and Supplemental Figure 4). Clusters 1–3, abundantly expressing fibroblast markers such as *PDGFRA, PDPN, FAP,* or *PRG4*, were characterized as synovial fibroblasts (Figure 3A and Supplemental Figure 4). We annotated the 3 synovial fibroblast clusters as *CD34^lo^THY1^lo^*, *CD34^lo^THY1^hi^*, and *CD34^hi^* subsets, (clusters 1, 2, 3, respectively), according to the previous reports of human RA synovium (Figure 3A and Supplemental Figure 4) (8). *CD34^lo^THY1^lo^* cells, most abundantly expressing *PRG4*, are lining layer fibroblasts, while *CD34^lo^THY1^hi^* and *CD34^hi^* are sublining layer subsets (8, 11). Clusters 6 and 7 were identified as macrophages, and cluster 8 as DCs (Figure 3A and Supplemental Figure 4). Macrophages were further divided into 2 clusters: a *MERTK^hi^CD206^hi^* subset, which was abundant in normal or remission RA synovium, and a *MERTK^lo^CD206^lo^* subset, which was abundant in inflammatory phase RA synovium (Figure 3A) (9). We next evaluated the proportions of synovial cell subsets among the 4 OA subgroup samples. Among the immune cells, macrophages were the most dominant both in the inflammatory and fibrotic types (Figure 3, B and C). The percentage of *MERTK^lo^CD206^lo^* macrophages was higher in the inflammatory type samples and lowest in the Fibro_2 sample (Figure 3, B and C). T cells were the most abundant in the Fibro_2 sample. As for fibroblasts, the rates of *CD34^hi^* and *CD34^lo^THY1^lo^* fibroblasts were highest and lowest in Fibro_2 sample, respectively (Figure 3, B and C).

In the present OA synovium data, *MERTK^lo^CD206^lo^* macrophages more abundantly expressed various chemokines, which are associated with leukocyte recruitment and activation, compared with the *MERTK^hi^CD206^hi^* subset (Figure 3D). We further characterized the 3 fibroblast subsets. *CD34^lo^THY1^lo^* fibroblasts showed expression of catabolic enzyme genes such as *MMP*s (Figure 3E). *CD34^lo^THY1^hi^* fibroblasts highly expressed inflammatory cytokines and chemokines such as *CCL2* and *IL6* (Figure 3E). *CD34^lo^THY1^hi^* fibroblasts also expressed an angiogenesis-related gene *ANGPT1* (Figure 3E). Meanwhile, genes associated with fibrosis and angiogenesis were highly expressed in *CD34^hi^* fibroblasts (Figure 3E). Notably, marker genes for universal fibroblasts, including *PI16*, *COL15A1*, and *DPT* (12), were highly expressed in *CD34^hi^* fibroblasts (Figure 3F). BMPs and WNT antagonists were also highly expressed in *CD34^hi^* fibroblasts (Figure 3G). GO terms related with extracellular matrix organization, fibrosis, and angiogenesis were more prevalent in *CD34^hi^* fibroblasts, compared with other 2 subsets (Figure 3H).

**Figure 4:**
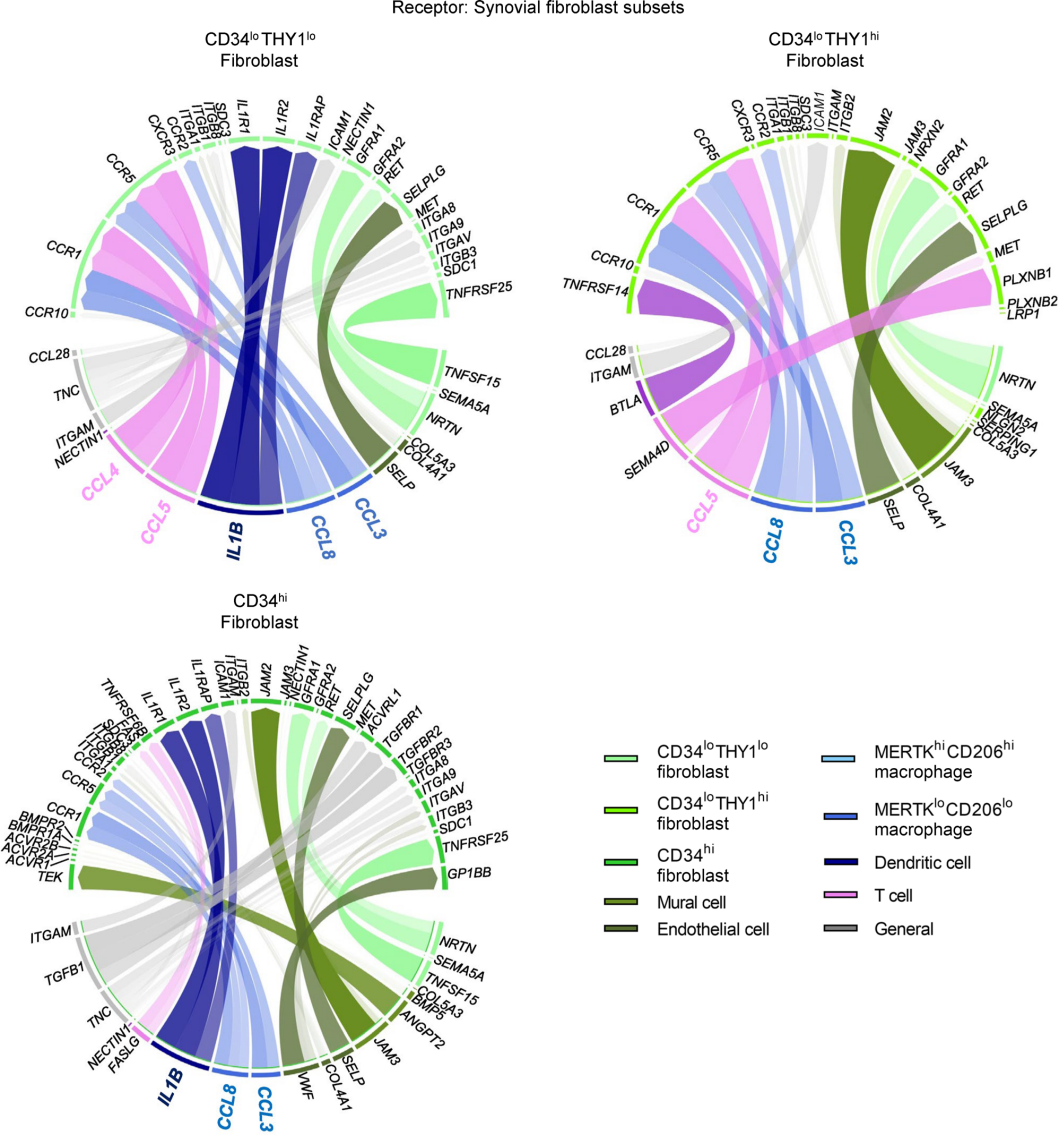
Estimated interactions between synovial cell subsets when three fibroblast subsets are set as the receptors. Ligand-receptor analyses setting each of 3 fibroblast subsets (*CD34^lo^THY1^lo^*, *CD34^lo^THY1^hi^*, and *CD34^hi^* fibroblasts) as recipients. The top ligands observed in at least 2 clusters were described as General.

**Figure 5:**
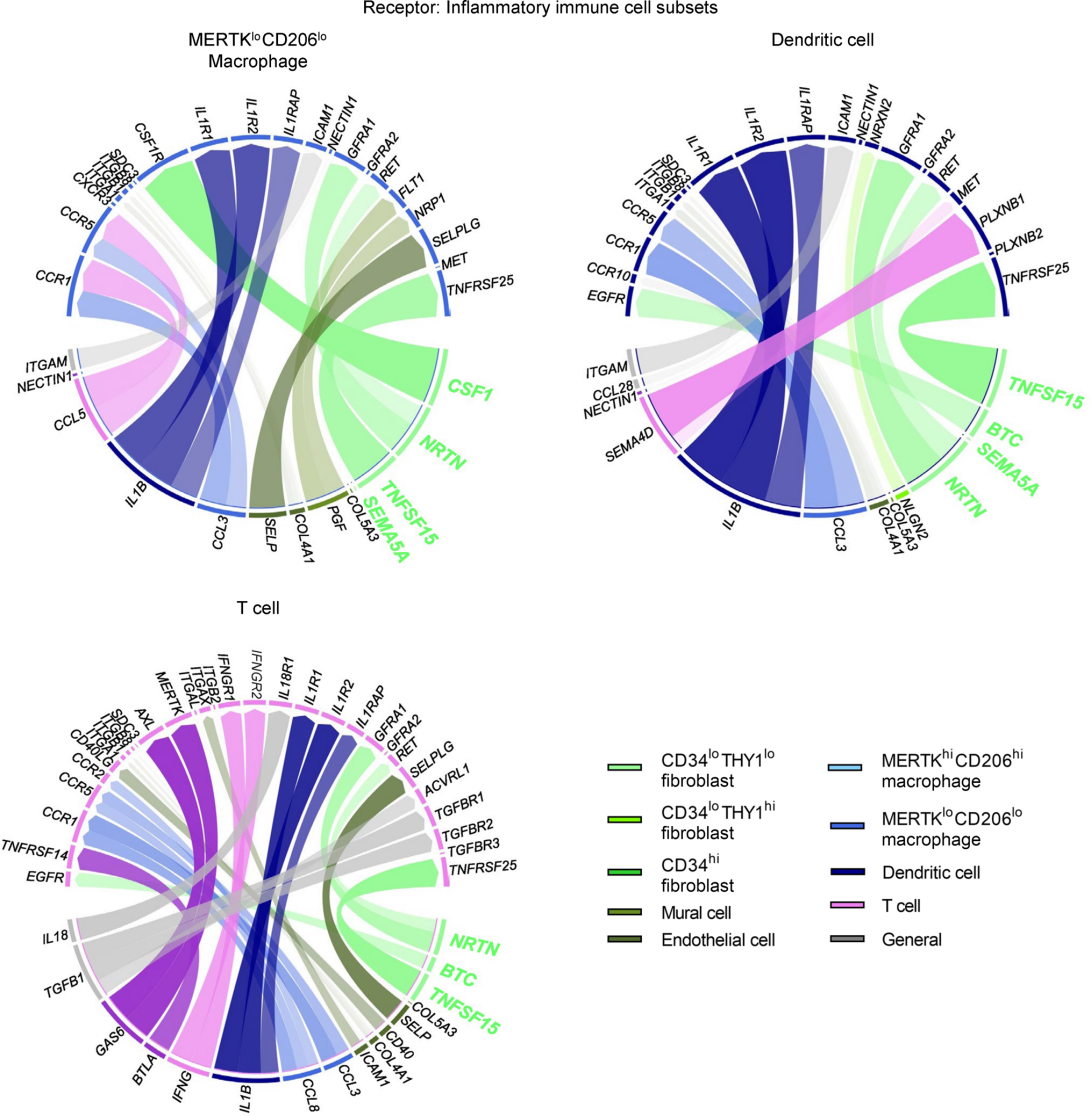
Estimated interactions between synovial cell subsets when inflammatory immune cell subsets are set as the receptors. Ligand-receptor analyses setting each of 3 immune cell subsets (*MERTK^lo^CD206^lo^* macrophages, DCs, and T cells) as recipients. The top ligands observed in at least 2 clusters were described as General.

Cross-talk interactions between the synovial cell clusters were estimated by ligand-receptor analyses. When we set the fibroblast subsets as recipients, vigorous interactions from immune cells (such as T cells, macrophages, and DCs) to *CD34^lo^THY1^lo^* fibroblasts were prominent, such as *CCL3*, *CCL4*, *CCL5*, *CCL8*, and *IL1B* (Figure 4). In contrast, as for *CD34^hi^* fibroblasts, interactions from immune cells were modest, while those from mural and endothelial cells were prominent instead (Figure 4). Ligand-receptor analyses with immune cells as recipients detected several interactions from *CD34^lo^THY1^lo^* fibroblasts (Figure 5). *NRTN*, *BTC*, and *SEMA5A* were detected from *CD34^lo^THY1^lo^* fibroblasts to immune cells, as were essential cytokines for macrophage regulation such as *CSF1* and *TNFSF15* (Figure 5). On the other hand, secreted molecules for the immune cells were not detected from *CD34^hi^* fibroblasts (Figure 5). Overall, these data imply that *CD34^lo^THY1^lo^* fibroblasts interact most actively with immune cells, while *CD34^hi^* fibroblasts show the least interaction.

### Interactions between synovial cell clusters and articular chondrocytes.

**Figure 6:**
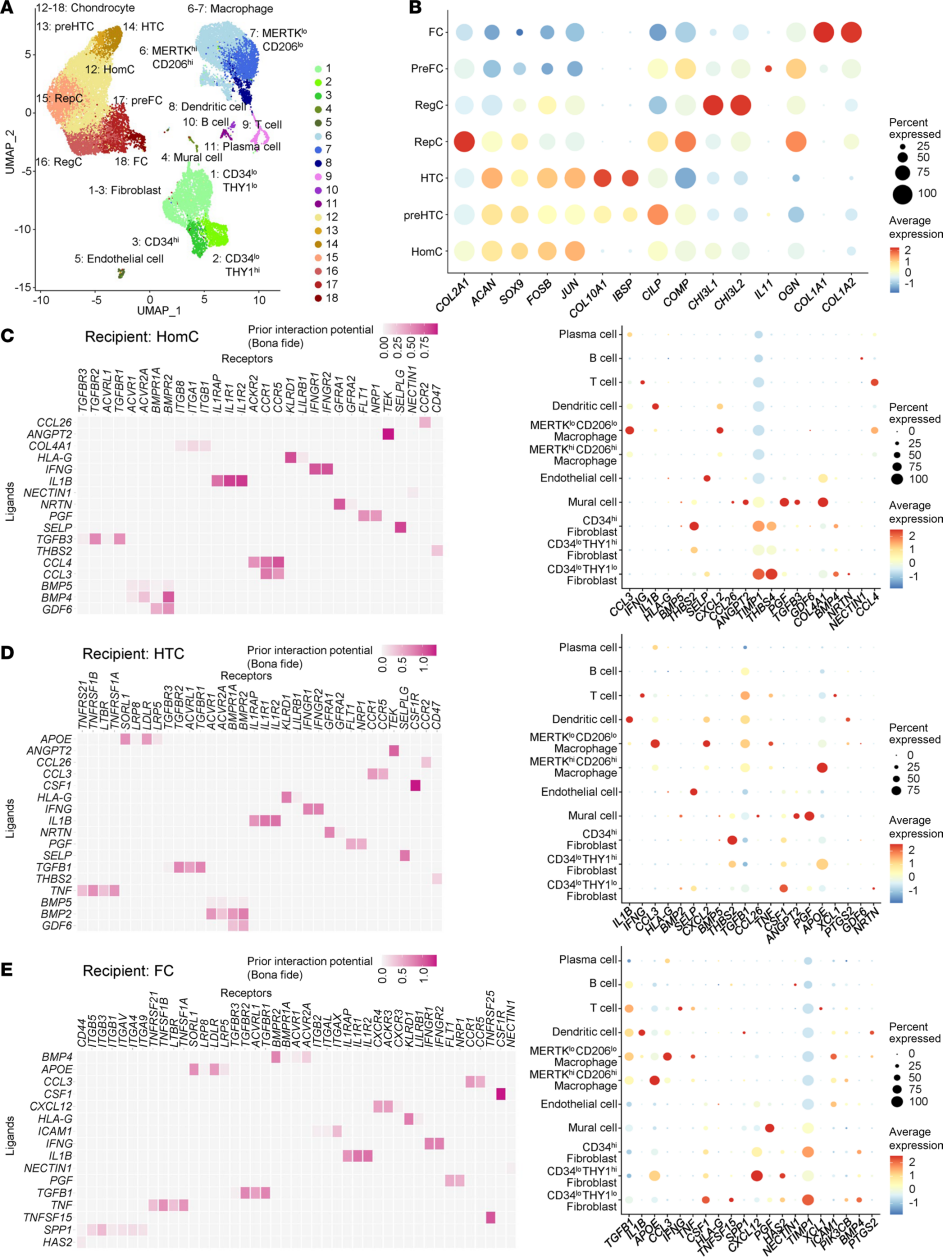
Estimated interactions between synovial cell subsets and articular chondrocytes using the merged datasets. (**A**) UMAP of 18 synovial cell and chondrocyte clusters identified by scRNA-Seq. HomC, homeostatic chondrocyte; preHTC, prehypertrophic chondrocyte; HTC, hypertrophic chondrocyte; RepC, reparative chondrocyte; RegC, regulatory chondrocyte; preFC, prefibrotic chondrocyte; FC, fibrotic chondrocyte. (**B**) Dot plots of representative marker genes in 7 chondrocyte clusters. (**C**–**E**) Ligand-receptor analyses between each of 3 representative chondrocyte subsets and all synovial cell and chondrocyte subsets. Each of the 3 chondrocyte subsets — i.e., homeostatic (**C**), hypertrophic (**D**), and fibrotic (**E**) — was set as the recipient. Left: heatmaps of ligand-receptor interaction potential. Right: dot plots of the top 20 ligand gene expression profiles.

We further estimated interactions between synovial cells and articular chondrocytes using the present and deposited scRNA-Seq datasets. We merged our knee OA synovium scRNA-Seq dataset with a deposited knee OA articular chondrocyte scRNA-Seq dataset (GSE152805) (13). When the 2 datasets were merged and batch effects were removed, the same clusters were identified again: 3 fibroblast subsets, 2 macrophage subsets, and other immune cells of the synovial cell population (Figure 6A). Homeostatic, prehypertrophic, hypertrophic, reparative, regulatory, prefibrotic, and fibrotic chondrocytes were also identified from the chondrocyte population, according to the original study (Figure 6, A and B) (13). We then analyzed the top ligands expressed in synovial cells by setting homeostatic, hypertrophic, or fibrotic chondrocyte subsets as recipients. One of the prominent inflammatory cytokines, *IL1B*, was exclusively expressed in DCs and *MERTK^lo^CD206^lo^* macrophages (Figure 6, C–E). *TNF* was mainly expressed in *MERTK^lo^CD206^lo^* macrophage and was found to influence hypertrophic and fibrotic chondrocytes (Figure 6, D and E). On the other hand, fibroblasts expressed different kinds of ligand genes. *BMP4*, an anabolic cytokine for chondrocytes (14–16), and *TIMP1*, a potent inhibitor for MMP (17, 18), were highly expressed in *CD34^hi^* and *CD34^lo^THY1^lo^* fibroblasts (Figure 6, C and E). Notably, *THBS2*, coding thrombospondin-2, was intensively detected from *CD34^hi^* fibroblasts to homeostatic and hypertrophic chondrocytes (Figure 6, C and D). Many studies have indicated that expression of thrombospondin-2 is elevated in OA and that thrombospondin-2 promotes chondrogenesis and inhibits hypertrophic differentiation of chondrocytes (19). *CD34^lo^THY1^hi^* fibroblasts abundantly expressed *APOE* and *CXCL12* for hypertrophic and fibrotic chondrocytes, respectively (Figure 6, D and E). APOE, encoded by *APOE*, may be associated with OA development (10). Interestingly, *CSF1* from *CD34^lo^THY1^lo^* fibroblasts to *CSF1R* in hypertrophic and fibrotic chondrocytes were detected (Figure 6, D and E). Similar molecules were detected through the same analyses, setting other chondrocyte subsets as recipients (Supplemental Figure 5, A–D).

### Identification of CD34^hi^CD70^hi^ fibroblasts.

**Figure 7:**
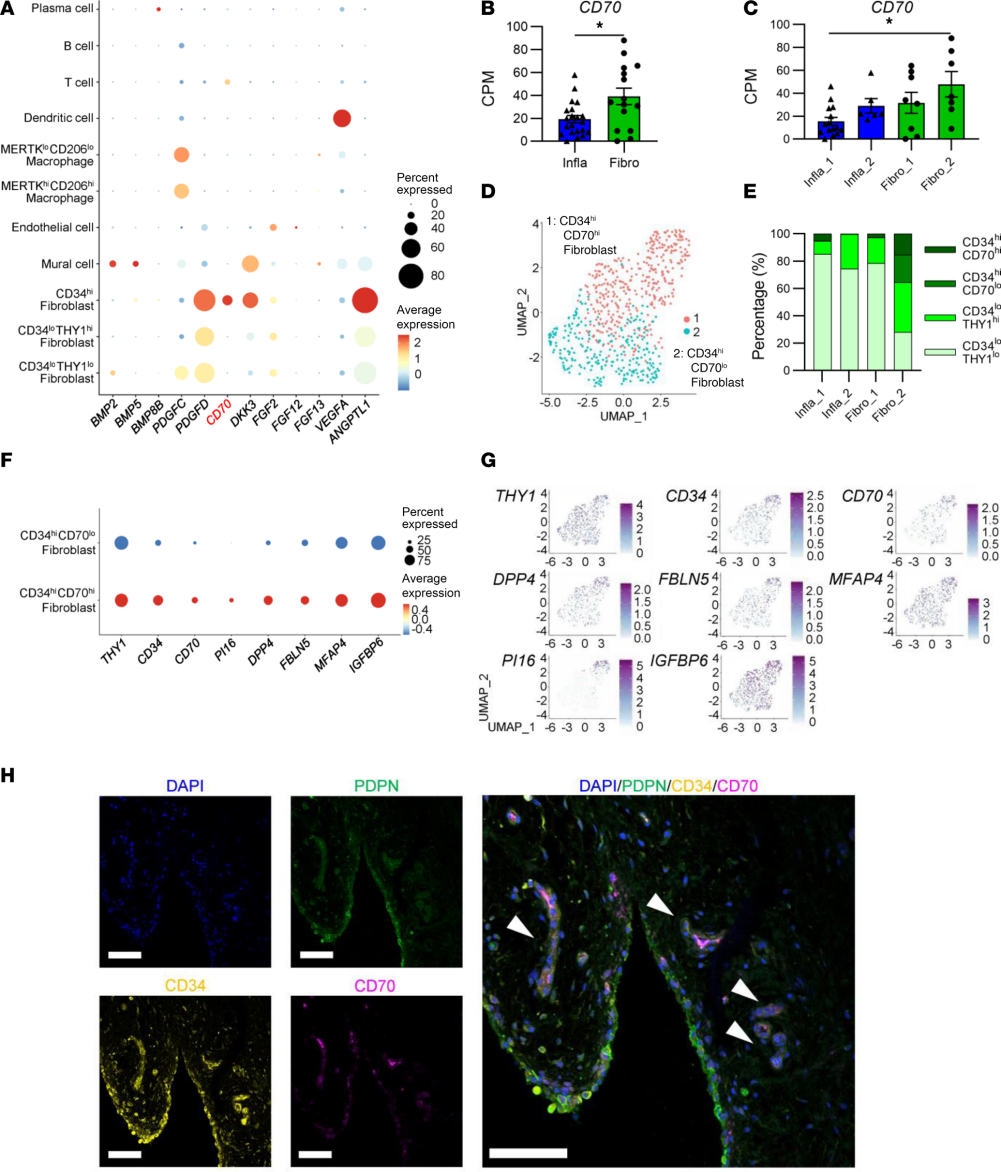
The expression profile of CD70 in OA synovium, especially fibroblast. (**A**) Dot plots of representative DEGs from fibrotic group synovium (Figure 1C) in 11 synovial cell subsets (Figure 3). (**B**) Expression levels of CD70 in 2 OA synovium groups, determined by knee OA synovium RNA-Seq. All data are expressed as dot plots and mean ± SEM. Mann-Whitney *U* test. *P < 0.05. (**C**) Expression levels of CD70 in 4 OA synovium subgroups, determined by knee OA synovium RNA-Seq. All data are expressed as dot plots and mean ± SEM. Kruskal-Wallis test. *P < 0.05. (**D**) UMAP of 2 *CD34^hi^* fibroblast clusters identified by scRNA-Seq. (**E**) Percentage of fibroblast subsets in the 4 synovium samples. Infla, inflammatory group; Fibro, fibrotic group. (**F**) Dot plots of stable state marker genes and CD70 in *CD34^hi^CD70^lo^* fibroblasts and *CD34^hi^CD70^hi^* fibroblasts. (**G**) Feature plots of stable state marker genes and CD70 in *CD34^hi^CD70^lo^* fibroblasts and *CD34^hi^CD70^hi^* fibroblasts. (**H**) Immunofluorescence images of Podoplanin (PDPN), CD34, and CD70 in human knee OA synovium tissue around vessels. Arrowheads indicate PDPN^+^CD34^+^CD70^+^ cells. Scale bar: 100 μm.

The results suggest that the *CD34^hi^* fibroblast subset showed least interaction with immune cells via proinflammatory cytokines and may also act protectively against cartilage. Among the cytokines, chemokines and growth factors with high expression levels in the bulk RNA-Seq fibrotic type — *BMP5*, *BMP8B*, *PDGFD*, *DKK3*, and *CD70 —* were also highly expressed in the scRNA-Seq *CD34^hi^* fibroblast subset (Figure 7A). Although several protective effects on chondrocytes have been reported in the past for the BMP family, the *PDGF* family, and WNT inhibitors (14–16, 20–22), there are no previous reports for *CD70*, a member of the *TNF* superfamily molecules. CD70 is expressed in lymphocytes and usually delivers signals to T cells through CD27 (23, 24). Although many studies have indicated the pathological roles of the CD70/CD27 axis in RA (24), those in OA or effects of CD70 expressed in synovial fibroblasts have not been revealed. Thus, we decided to focus on function of CD70 in knee OA synovium. In the present RNA-Seq data, CD70 was highly expressed in the fibrotic group, especially in the Fibro_2 group (Figure 7, B and C). *CD34^hi^* fibroblasts were divided to *CD70^lo^* and *CD70^hi^* subsets (Figure 7D). In our scRNA-Seq data of OA synovium, the percentage of *CD34^hi^CD70^hi^* fibroblasts was highest in the Fibro_2 sample (Figure 7E). *CD34^hi^CD70^hi^* fibroblasts expressed marker genes for universal fibroblasts, including *PI16* and *DPP4* (12) (Figure 7, F and G). In multiple immunofluorescence staining, CD70^+^ cells were observed in some of the CD34^+^ fibroblasts (PDPN^+^CD34^+^ cells), mainly around vessels (Figure 7H).

### CD34^hi^CD70^hi^ fibroblasts are involved in regulatory T cell proliferation.

**Figure 8:**
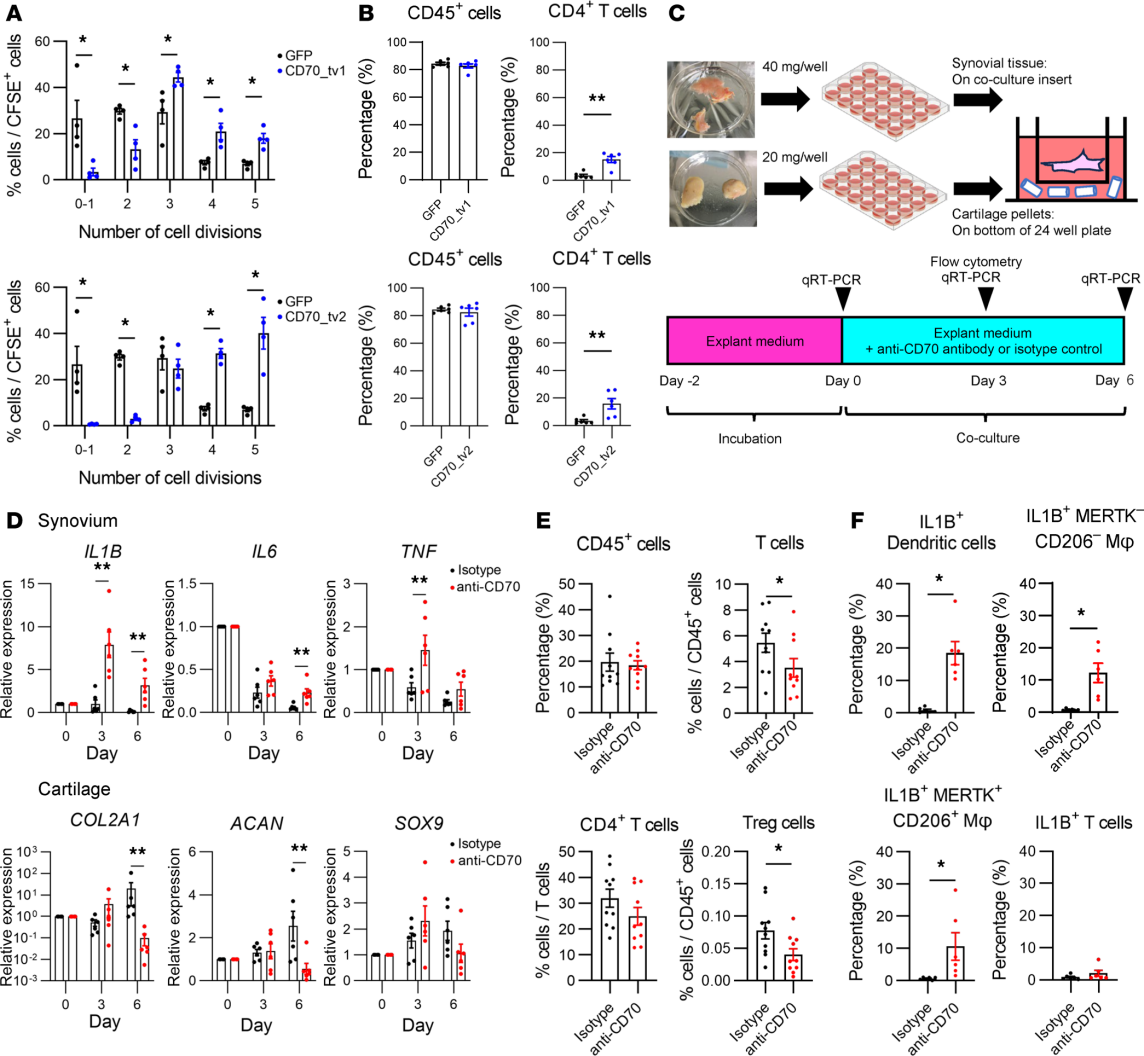
CD70 is crucial for T cell proliferation and contributes to suppression of synovitis by Tregs. (**A**) T cell proliferation assay cocultured with CD70 transcript variant 1 or 2 or with GFP-transfected fibroblasts cell line (MRC-5). (**B**) The percentage of CD45^+^ cells and CD4^+^ T cells in live cells cocultured with CD70 transcript variant 1 or 2 or with GFP-transfected MRC-5 cells. (**C**) Graphical schema of OA synovial and cartilage tissues coculture experiments. (**D**) Time-course mRNA levels of *IL1B*, *IL6*, and *TNF* in cocultured synovial tissues and *COL2A1*, *ACAN*, and *SOX9* mRNA levels in cocultured cartilage tissues with anti-CD70 antibody or isotype control. *n* = 6 biologically independent experiments. (**E**) The percentage of CD45^+^ cells and T cell subsets in cocultured synovial tissues with anti-CD70 antibody or isotype control. *n* = 10 biologically independent experiments. (**F**) The percentage of IL-1B^+^ DCs, macrophages (Mφ), and T cells in cocultured synovial tissues with anti-CD70 antibody or isotype control. *n* = 6 biologically independent experiments. Data are expressed as dot plots and mean ± SEM. Mann-Whitney *U* test. *P < 0.05, **P < 0.01.

The CD70/CD27 axis is involved in the differentiation and proliferation of T and B cells (23, 24). In the current RNA-Seq data, T cells were the most abundant in the Fibro_2 sample, as were *CD34^hi^* fibroblasts (Figure 3C). To test the effect of CD70-expressing fibroblasts on T cells, we cocultured fibroblast cell line MRC-5, transfected with an expression vector of CD70 transcript variant 1 or 2 and CD4^+^ T cells sorted from healthy volunteer peripheral blood. Coculture with CD70-transfected MRC-5 cells significantly increased the number of CD4^+^ T cell divisions and its percentage in viable cells compared to coculture with GFP-transfected fibroblasts (Figure 8, A and B). Next, we performed organ culture to examine the interaction between human knee OA synovium and cartilage tissue under antibody blockade of CD70 (Figure 8C). Anti-CD70 antibody treatment increased mRNA levels of *IL1B*, *IL6*, and *TNF* in OA synovial tissues and decreased those of *COL2A1*, *ACAN*, and *SOX9* in OA cartilage tissues (Figure 8D). When the anti-CD70 antibody was added to organ cultures of cartilage tissue alone, no significant changes were observed in these cartilage markers (Supplemental Figure 6). Given that *CD27*, the only receptor for *CD70* (23), is mainly expressed on T and B cells, it is presumed that anti-CD70 antibody treatment enhanced the expression of inflammatory cytokines in immune cells of synovial tissue and suppressed chondrocyte differentiation. The scRNA-Seq data indicate that the percentage of Tregs among the synovial immune cells was higher in the Fibro_2 group compared with the other groups (Supplemental Figure 7). We finally examined the effect of CD70 inhibition on T cells by flow cytometry, using cells collected from synovial tissue after the treatment of anti-CD70 antibody or isotype control for 3 days in the coculture model described before. The anti-CD70 antibody treatment did not change the percentage of leukocytes among viable cells in synovial tissues; however, it significantly decreased the percentage of T cells, particularly Tregs among leukocytes (Figure 8E). Furthermore, the rates of IL-1B^+^ macrophages and DCs significantly increased by the anti-CD70 antibody treatment (Figure 8F). These data suggest that *CD34^hi^* fibroblasts may attenuate activation of macrophages and DCs through the CD70/CD27 axis and that Tregs may be one of the target cells of CD70 expressed in *CD34^hi^* fibroblasts.

## Discussion

The present study demonstrated 2 types of knee OA with different clinical manifestations according to the status of the synovium. The 2 groups, determined by RNA-Seq of OA synovial tissues, were characterized as inflammatory or fibrotic according to their gene expression profiles and histological findings. Joint pain scores in the inflammatory group were significantly worse than those in the fibrotic group. scRNA-Seq displayed 3 fibroblast subsets and 2 macrophage subsets, consistent with previous studies. The *MERTK^lo^CD206^lo^* macrophages and *CD34^hi^* fibroblasts were dominant in the inflammatory and fibrotic types, respectively. The scRNA-Seq data also indicate that the *CD34^lo^THY1^lo^* and *CD34^lo^THY1^hi^* fibroblasts might be affected by synovial immune cells such as T cells, DCs, and macrophages, while *CD34^hi^* fibroblasts might be affected by mural and endothelial cells. In addition, we estimated the effects of these synovial cells on articular chondrocytes by merging our data with the deposited scRNA-Seq data of human OA cartilage. Finally, we demonstrated that *CD34^hi^CD70^hi^* fibroblast are possibly crucial for Treg proliferation suppressing knee OA synovitis.

Recent studies using scRNA-Seq have revealed the characteristics of synovial fibroblasts responsible for RA pathophysiology (8, 9, 11, 25). However, the reports on synovial cell subsets responsible for OA pathophysiology or phenotype have been limited (26–28). In this study, the *CD34^lo^THY1^lo^* and *CD34^lo^THY1^hi^* subsets of OA synovium displayed high similarity to their counterpart subsets in previous datasets (Figure 3, D and E). On the other hand, the expression level of *RANKL*, which was reported to be higher in the CD34^−^THY1^+^ subset of RA synovium (8), was lower in the *CD34^lo^THY1^hi^* and another 2 subsets of OA fibroblasts (Figure 3E). Considering that *RANKL* is an osteoclastogenesis-inducing factor, the difference in *RANKL* expression may be involved in the bone phenotypes of both diseases. Similarly, *CD34*^+^ fibroblasts in RA were reported to more highly express inflammatory cytokines (including *CCL2* and *IL6*) than other subsets and have a role in leukocyte recruitment (8). However, in this study, the *CD34^hi^* subset showed fibrotic and stable characteristics, expressing matrix proteins such as *FBN1*, as well as *FBLN1,* BMPs, and WNT pathway inhibitors (Figure 3, E–G). The *CD34^hi^* fibroblasts showed circulation and fibrosis-related GO terms (Figure 3H) and were more affected by mural and endothelial cells rather than by immune cells (Figure 4). Perivascular fibrosis was observed in fibrotic group synovium, which includes more *CD34^hi^* fibroblasts. Considering these findings and the evidence for CD34 as a common marker for diverse progenitors (29), the *CD34^hi^* subset may alleviate the pathogenesis of OA.

In addition to fibroblasts, immune cells also play essential roles in the synovium. Alivernini et al. showed that MERTK^+^CD206^+^ and MERTK^–^CD206^–^ macrophages have tissue-resolving and proinflammatory characteristics, respectively (9). The present findings regarding *MERTK^hi^CD206^hi^* and *MERTK^lo^CD206^lo^* macrophages in OA synovium are similar to the previous ones. Proinflammatory cytokines from the immune cells contribute to proliferative, angiogenic, and catabolic features of fibroblasts (30, 31). In our study, inflammatory *MERTK^lo^CD206^lo^* macrophages were more prominent in the inflammatory group than in the fibrotic group (Figure 3, A–C). The ligand-receptor analyses suggest that these *MERTK^lo^CD206^lo^* macrophages actively regulate *CD34^lo^THY1^lo^* and *CD34^lo^THY1^hi^* fibroblasts (Figure 4). Similar to RA, *MERTK^lo^CD206^lo^* macrophages are probably involved in the pathophysiology of OA.

Finally, we estimated the role of *CD34^hi^CD70^hi^* synovial fibroblasts. Mizoguchi et al. reported that CD34^+^ fibroblasts are the subset that highly express IL-6 and have high monocyte migration potential (8). On the other hand, Noda et al. reported that CD34^+^THY1^+^ fibroblasts exhibit high osteoblast and chondrocyte differentiation potential (32), thus whether this CD34^+^ subset promotes or inhibits the pathogenesis of OA and RA has been left unresolved. CD70 activates T cell proliferation through the CD70/CD27 axis, which has been reported to both promote and suppress inflammation. CD70^+^CD4^+^ T cells have been reported to increase in many autoimmune diseases, including systemic lupus erythematosus and multiple sclerosis (33), while CD70^+^ cancer-associated fibroblasts have been reported to promote proliferation of Tregs and create an environment to evade host immunity (34). In the present study, the treatment of anti-CD70 antibody decreased the proportion of Tregs and increased the proportion of IL-1B^+^ monocytes in immune cells of synovial tissue, and it exacerbated synovitis (Figure 8, D–F). These results suggest that C*D34^hi^CD70^hi^* fibroblasts may restrain synovitis in knee OA via T cell proliferation including Tregs and also have an aspect of relieving OA pathophysiology. These roles of C*D34^hi^CD70^hi^* fibroblasts should be further investigated by in vivo mechanistic studies.

Needless to say, inflammatory and fibrotic groups are overlapping, and the overall data display gradient distribution accompanied by samples of intermediate characters (Figure 2B). Heberden nodes, OA of distal interphalangeal finger joints, display 2 phases: an inflammatory phase first and a subsequent ankylosing phase (35). The inflammatory and fibrotic types shown in this study may also represent the 2 typical phases of finger OA. In the present study, we could not obtain normal synovial tissues, and we did not conduct scRNA-Seq of the synovial samples from arthroscopic surgery. Comparison with more healthy samples would be helpful for further understanding the association of synovial cell types and joint phenotypes as well as OA pathophysiology.

Recently, a series of studies have reported promising effects of intraarticular administration of mesenchymal stem cells for knee OA, although further improvements are needed (36). The present findings regarding C*D34^hi^* fibroblasts may contribute to enhancing stem cell therapy. Additionally, many emerging therapies using chemical compounds or antibodies for OA have also been investigated in clinical trials (37). The inflammatory and fibrotic types in this study may be suited for different types of treatment, and in the future, less-invasive technology to survey the phenotype of knee OA will be required.

In conclusion, the present study revealed inflammatory and fibrotic groups of OA synovium, depending on their gene expression profiles. These groups are associated with joint pain and are characterized by different kinds of component synovial cells. *CD34^hi^CD70^hi^* fibroblasts potentially relieve knee OA synovitis by proliferation of Tregs. Elucidation of the mechanisms underlying the regulation of these various synovial cells may lead to novel strategies for OA therapeutics.

## Methods

### Sex as a biological variable

Both male and female patient samples were used in this study. There were no statistically significant differences in the sex ratio between the groups, as shown in Supplemental Table 1.

### Participants and synovial tissue collection

Patients aged ≥ 20 years were recruited, but patients using immunosuppressants or anticancer agents were excluded. We recruited 36 patients with OA undergoing initial TKA and 5 patients undergoing knee arthroscopy about 1 year after anterior cruciate ligament reconstruction. The suprapatellar synovial tissues of all patients were submitted to bulk RNA-Seq and utilized for histological evaluation.

### Synovial tissue processing

Synovial tissue processing was performed according to the protocol reported by Donlin et al. (8, 11, 38). Immediately after collection, surgical specimens were removed from bone and adipose tissues using surgical scissors, and the remaining synovial tissues were cut into smaller pieces before enzymatic treatment. For scRNA-Seq sample preparation, synovial tissues that had been cryopreserved by CryoStor CS10 (HemaCare Corporation) at –80°C were thawed in RPMI-1640 medium and digested in RPMI-1640 medium containing 0.2 mg/mL Liberase TL (Roche) and 0.1 mg/mL DNase I (Nakarai Tesque) for 30 min at 37°C. After enzymatic treatment, the cell suspension was centrifuged (150*g* for 3 minutes) and washed, and erythrocytes were lysed using RBC Lysis Buffer (10×) (BioLegend). For RNA-Seq sample preparation, synovial tissues from the suprapatellar site were crushed with a bead homogenizer, dissolved in TRI Reagent (Molecular Research Center), and stored at –80°C until sample submission. For flow cytometry, we used nonfrozen tissues, and there was no thawing process in sample preparation.

### Histological evaluation of synovial tissue

A part of collected synovial tissues was fixed overnight in 10% buffered formalin. After dehydration and defatting, tissues were paraffin embedded, and sections were prepared at a thickness of 4 μm. The specimens were utilized for H&E and Masson’s trichrome staining. For specimens undergoing H&E staining, 2 expert examiners blindly assessed the presence or absence of inflammatory cell infiltration as reported by Krenn et al. (39) and formed a consensus. For specimens undergoing Masson’s trichrome staining, positive areas were quantitatively assessed using Aperio Image Scope (v12.3.3 Leica) in a representative area, including a synovial lining layer with 4,000 μm width and 2,000 μm depth.

### RNA-Seq

Total RNA was extracted from fresh synovial specimens using Direct-zol RNA MicroPrep Kit (Zymo Research), and the library was prepared using the NEBNext Ultra II RNA Library Prep Kit (Illumina) according to each specimen protocol. Sequencing was performed by NovaSeq 6000 (Illumina) with two 150 bp paired-end reads and about 10 million reads per sample, on average.

### Bioinformatic analysis of RNA-Seq data

Before data analysis, quality control was performed on FastQC data using fastp (v0.20.T), and RNA-Seq reads were mapped to GENECODE v37 (GRCh38.p13) using STAR (v2.7.3a). Then, transcript and gene expression levels were quantified using RMSE (v1.3.3). The read count data were exported to CSV file, and subsequent analysis was performed using iDEP (v0.93) (40). Counts data were filtered to genes whose expression levels were greater than 0.5 counts per million (CPM) in at least 1 sample, and then edgeR was used for normalization. In the hierarchical clustering, the top 500 genes with high expression variation were used. DEGs with FDR of 0.1 or less and the minimal fold change of 2 or more between the groups were extracted using DESeq2. Pathway analysis was performed using gene set enrichment analysis (GSEA).

### scRNA-Seq

Cryopreserved synovial tissues were enzymatically treated using the protocol described previously to obtain a single-cell suspension and resuspended at a concentration of 1,000 cells/μL. Cell viability, confirmed at approximately 80%, was assessed using the trypan blue exclusion method with automated counting by LUNA-FL (Logos Biosystems). Then, the cell suspension was prepared according to the protocol of Chromium Next GEM Single Cell 3′ Reagent Kits (v3.1 10X Genomics), and a single cell emulsion was produced using Chromium Controller (10X Genomics). Each library was sequenced using NovaSeq 6000 (Illumina) with 28 or 91 bp paired-end reads and about 50,000 reads per cell on average.

### Bioinformatic analysis of scRNA-Seq data

#### Processing raw reads.

scRNA-Seq reads were mapped using reference genome hg38:refdata-gex-GRCh38-2020-A provided by 10X Genomics with default parameters according to Cell Ranger pipeline (v5.0.0) and a unique molecular identifier (UMI) count matrix was generated.

#### Quality control and filtering.

We created a Seurat object using R (v4.0.2) and the Seurat package (v4.0.0) (41, 42). Genes detected in a minimum of 3 cells and cells with a minimum of 200 genes detected were used to create the Seurat object. Then, cells with 1,600 or fewer expressed genes, and cells with 10% or more mitochondrial read content, were excluded from the data.

#### Merging datasets.

For the analysis of synovium, our 4 synovium datasets from 4 patients were merged. For the analysis of synovium and cartilage, our 4 synovium datasets and 6 cartilage datasets from NCBI Gene Expression Omnibus (GEO) GSE152805 (13) were merged. As for cartilage datasets, GSM4626766, GSM4626767, and GSM4626768 from the non-OA side and GSM4626769, GSM4626770, and GSM4626771 from the OA side were utilized. For the calculation simplicity, in the integrated analysis of synovium and cartilage datasets, the number of cells was reduced by random sampling before analysis (sampling rate < –0.5).

#### Normalization.

Cell cycle scores (S.Score, G2M.Score) of each cell were calculated using biomaRt (v2.46.3); then, sctransform (v0.3.2) was used to normalize the data. S.Score, G2M.Score, the percentage of mitochondrial read content(percent.MT), and sample ID of each cells were used as variables.

#### Dimensionality reduction and clustering.

Using the results of principal component analysis (PCA), dimensionality reduction was performed using the top 20 principal components (PCs) with reference to the Elbow Plot results. Clustering was performed using Clustree (v0.4.3) with the Leiden (v0.3.7) algorithm. The resolution was set so that the clusters were most stable. The resolution was set to 1.2 for our merged synovium dataset and to 1.2 for the merged synovium and cartilage dataset.

#### Annotation.

We annotated each cluster using marker genes of each cell type. In particular, the subsets of synovial fibroblasts, macrophages, and chondrocytes were annotated using previously reported marker genes (Figure 6B and Supplemental Figure 4) (8, 9, 11, 13, 43).

#### Additional analysis.

All genes with a log_2_ fold change of 0.25 or greater expressed in 10% or more of the cells in the cluster were extracted as variable genes using Seurat’s FindAllMarkers function. gprofiler2 (v0.2.0) was used to extract the top 20 GO biological process terms satisfying *P* < 0.05 for 3 clusters of fibroblasts. As for ligand-receptor analysis, nichenetr (v1.0.0) was used to extract the top 20 ligands for the receptor clusters, and circlize (v 0.4.13) was used for graphing.

### Immunofluorescence staining of synovial tissue

As for multiple immunofluorescence staining, sections were incubated with primary antibodies overnight at 4°C. Alexa Fluor–conjugated secondary antibodies were used, followed by DAPI containing an inclusion agent. To amplify the signal of CD70, tyramide signal amplification was used. Information on antibodies and reagents is provided in Supplemental Table 4. Fluorescence staining was evaluated by confocal microscopy, LSM880 (Carl Zeiss).

### Fibroblast cell line and CD4+ T cell coculture

The human fibroblast cell line, MRC-5 (RCB2833) was provided by the RIKEN BRC. MRC-5 cells were cultured in DMEM (low glucose) (Nakarai Tesque, 08456-65) with 10% FBS (Thermo Fisher Scientific, 10437-028) and 1% penicillin-streptomycin (Nakarai Tesque, 09367-34) and, after several passages, seeded in 24-well plates at 2.0×10^5^ cells/well. cDNA with CD70 transcript variant 1, CD70 transcript variant 2, or GFP cDNA was transfected into the cells using Lipofectamine 3000 (Invitrogen, L3000015). Next, CD4^+^ T cells collected from healthy volunteer peripheral blood using MidiMACS Separator (Miltenyi Biotec) and CD4^+^ T Cell Isolation Kit (Miltenyi Biotec, 130-096-533) were seeded at 5.0 ×10^5^ cells/well on 24-well plates, stimulated with 1 ng/mL αCD3 for 24 hours, and then transferred to wells of transfected fibroblasts. After 3 days of coculture, cells were collected and analyzed by flow cytometry.

### Synovial and cartilage tissue culture

Based on the report by Chan et al. (44), synovial and cartilage tissues of patients with knee OA were collected and cultured separately for 2 days in 24-well plates with 40 μg synovial and cartilage tissue in each well. The medium was a 1:1 mixture of RPMI 1640 (Nakarai Tesque, 30264-85), DMEM (low glucose) (Nakarai Tesque, 08456-65), and 1% FBS (Thermo Fisher Scientific, 10437-028), 1% penicillin-streptomycin (Nakarai Tesque, 09367-34), 1 mM sodium pyruvate (Nakarai Tesque, 29806-54), 50 μg/mL L-proline (Nakarai Tesque, 29001-71), 50 μg/mL L-ascorbic acid 2-phosphate (Nakarai Tesque, 13570-66), and 50 μg/mL 1× insulin-transferin-selenium (Thermo Fisher Scientific, 41400-045) were added. In the coculture experiments, after 2 days of culture, synovial and cartilage tissues were transferred to coculture plates, and each well was treated with anti–human CD70 antibody (NOVUS Bio, NBP3-11587) or isotype control (R&D Systems, MAB002) at a concentration of 5 μg/mL. The medium was changed after 3 days of coculture, and samples were collected at the start of coculture (Day 0), 3 days after coculture (Day 3), and 6 days after coculture (Day 6). Samples from Day 0, Day 3, and Day 6 were evaluated by quantitative PCR (qPCR), and samples from Day 3 by flow cytometry. For the organ culture of cartilage tissue alone, cartilage tissues were cultured with anti–human CD70 antibody or isotype control as we described above.

### Flow cytometry

Flow cytometry of synovial cells was performed using CytoFLEX S (Beckman Coulter). Cell numbers were counted using Karuza Analysis (v2.1 Beckman Coulter). In the evaluation of the percentage of immune cell subsets in synovial tissues (Figure 8, A, B, E, and F), 2 samples from 1 patient’s synovial tissue with similar macroscopic findings were used. For each experiment, 6–10 samples from 3–5 patients with knee OA were analyzed. The reagents used are listed in Supplemental Table 5.

### qPCR

Total RNA was extracted using the Direct-zol RNA Kit (Zymo Research, R2062) in accordance with the manufacturer’s protocol. Total RNA was reverse transcribed into cDNA using Rever Tra Ace qPCR RT Master Mix (TOYOBO, FSQ-201). qPCR was performed using THUNDERBIRD SYBR qPCR Mix (TOYOBO, FSQ-201) and Thermal Cycler Dice Real-Time System III (Takara Bio). All reactions were run in duplicate, and expression levels of genes were normalized to GAPDH expression level. Information on primers is given in Supplemental Table 6.

### Statistics

GraphPad Prism ver.9.1.2 was used for statistical analysis. Values are expressed as mean ± SD or mean ± SEM. For comparisons between the 2 groups, 2-tailed Student’s *t* tests were applied as parametric tests, and Mann-Whitney *U* tests were applied as nonparametric tests. For comparisons among more than 2 groups, Kruskal-Wallis test were applied. *P* < 0.05 were considered significant.

### Study approval

The protocol for this study was approved by the Ethics Committee of the University of Tokyo Hospital, Japan [review no. 0622-(12)], and written consent for the use of surgical specimens was obtained from all patients before surgery.

### Data availability

RNA-Seq and scRNA-Seq data have been deposited in the GEO (www.ncbi.nlm.nih.gov/geo/) under accession nos. GSE283079 and GSE283080, respectively. Values for all data points in graphs are reported in the Supporting Data Values file.

## Author contributions

JM, YO, RC, S Tanaka, and TS designed the research project. JM and RC collected synovial tissue samples with the cooperation of K Kono, K Kawaguchi, RY, H Inui, and S Taketomi. RC performed RNA-Seq. JM performed scRNA-Seq and other experiments. JM and RC conducted bioinformatics analysis on the advice of HO, MS, and YS. JM, AT, and TS performed figure editing. JM and TS wrote the manuscript with critical input from HO, H Ishikura, JH, NT, KN, S Tani, YI, AT, FY, RB, S Tanaka, and TS.

## Supplementary Material

Supplemental data

Supporting data values

## Version 1 01/23/2025

Electronic publication
