# Supplementary material for: CD34hi subset of synovial fibroblasts contributes to fibrotic phenotype of human knee osteoarthritis
Source: JCI Insight. 2025 Jan 23;10(2):e183690. doi: 10.1172/jci.insight.183690 (PMC11790023; doi:10.1172/jci.insight.183690)
Supplement: Supplemental data [file jciinsight-10-183690-s202.pdf]

Supplemental Figures and Tables

CD34^{high} subset of synovial fibroblasts contributes to fibrotic phenotype of human knee osteoarthritis

Junya Miyahara,[1] Yasunori Omata,[1,2] Ryota Chijimatsu,[2] Hiroyuki Okada,[1,3] Hisatoshi Ishikura,[1] Junya Higuchi,[1] Naohiro Tachibana,[1] Kosei Nagata,[1] Shoichiro Tani,[1,3] Kenichi Kono,[1] Kohei Kawaguchi,[1] Ryota Yamagami,[1] Hiroshi Inui,[1] Shuji Taketomi,[1] Yasuhide Iwanaga,[1,4] Asuka Terashima,[2] Fumiko Yano,[2] Masahide Seki,[5] Yutaka Suzuki,[5] Roland Baron,[6] Sakae Tanaka,[1]* and Taku Saito[1]*

[1]Sensory & Motor System Medicine, Graduate School of Medicine, The University of Tokyo, Tokyo, Japan.
[2]Bone and Cartilage Regenerative Medicine, Graduate School of Medicine, The University of Tokyo, Tokyo, Japan.
[3]Center for Disease Biology and Integrative Medicine, Graduate School of Medicine, The University of Tokyo, Tokyo, Japan.
[4]Department of Chemistry and Biotechnology, Graduate School of Engineering, The University of Tokyo, Tokyo, Japan.
[5]Laboratory of Systems Genomics, Department of Computational Biology and Medical Sciences, The University of Tokyo, Kashiwa, Japan.
[6]Department of Medicine, Harvard Medical School and Endocrine Unit, MGH, Boston, MA

*Corresponding authors

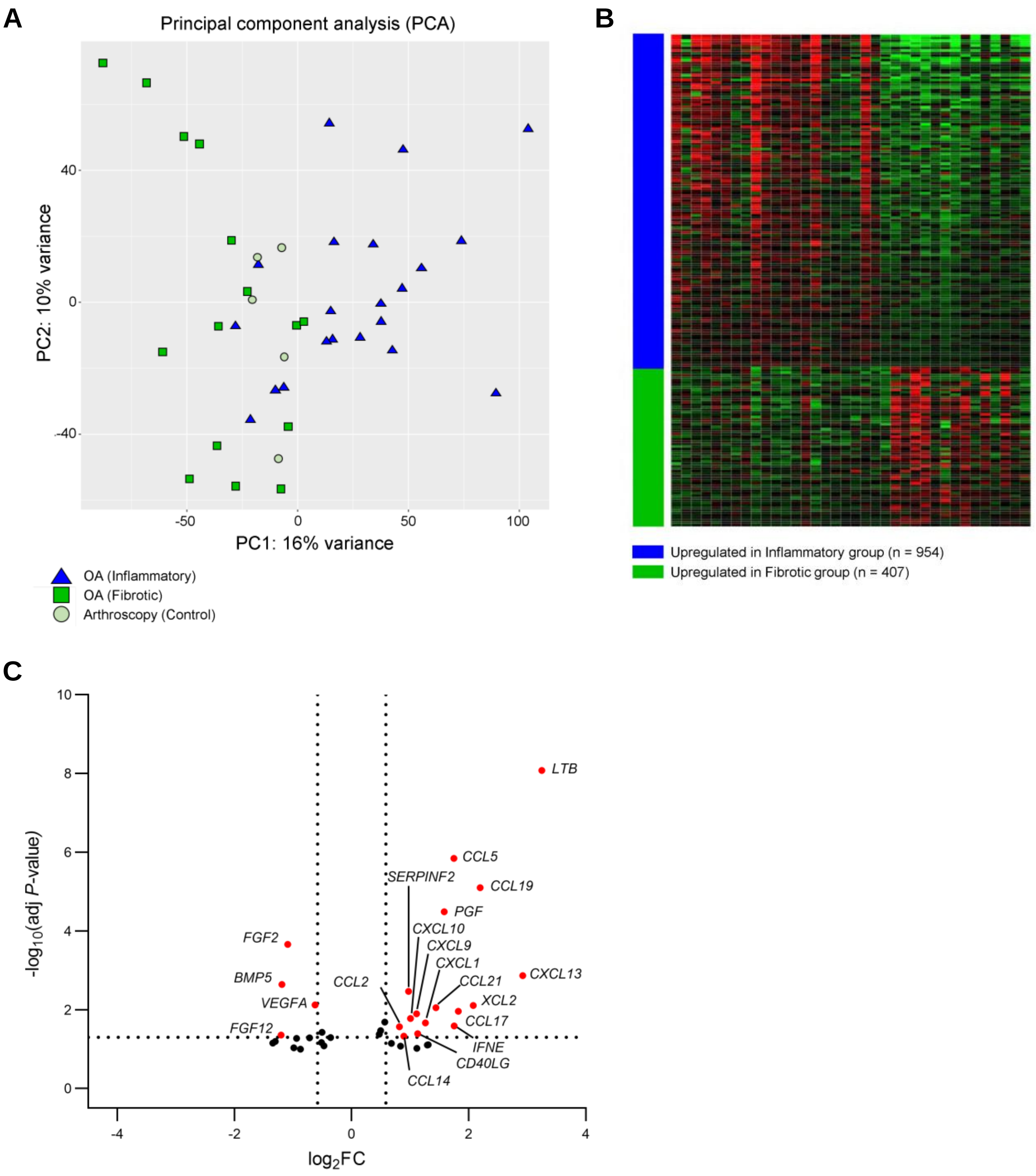

Supplemental Figure 1. Primary component analysis (PCA) and differentially expressed genes (DEGs) of human knee OA and arthroscopic surgery samples in RNA-seq. (A) PCA of 36 knee OA and 5 arthroscopic surgery patients synovium samples. Inflammatory group, n = 21; Fibrotic group, n = 15; Arthroscopic surgery group, n = 5. **(B)** DEGs in inflammatory and fibrotic groups. Inflammatory group, n = 21; Fibrotic group, n = 15. **(C)** Volcano plot of RNA-seq data comparing inflammatory with fibrotic type OA synovium. Each dot shows genes classified as cytokines, chemokines, and growth factors. Red dots show genes with |FC| > 1.5 and adjusted P value < 0.05.

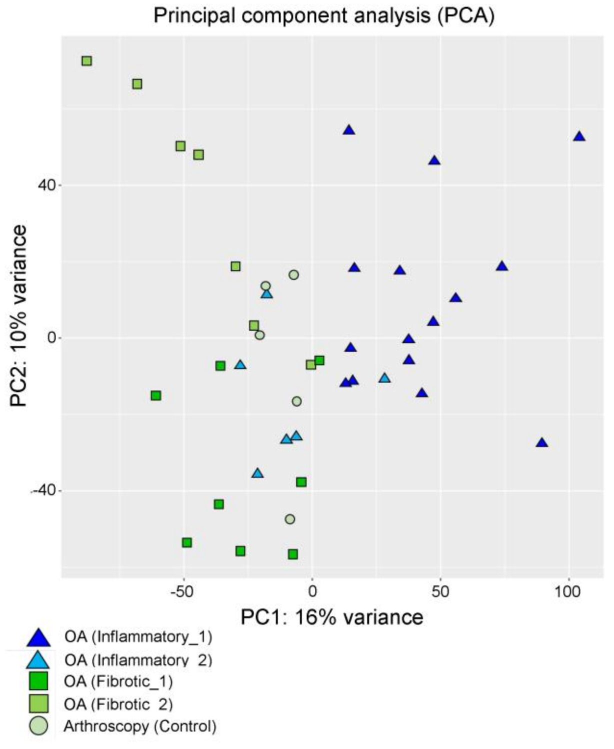

Supplemental Figure 2. Primary component analysis (PCA) of human knee OA and arthroscopic surgery samples in RNA-seq. PCA of 36 knee OA and 5 arthroscopic surgery patients synovium samples. Inflammatory_1 group, *n* = 15; Inflammatory_2 group, *n* = 6; Fibrotic_1 group, *n* = 8; Fibrotic_2 group, *n* = 7; Arthroscopic surgery group, *n* = 5.

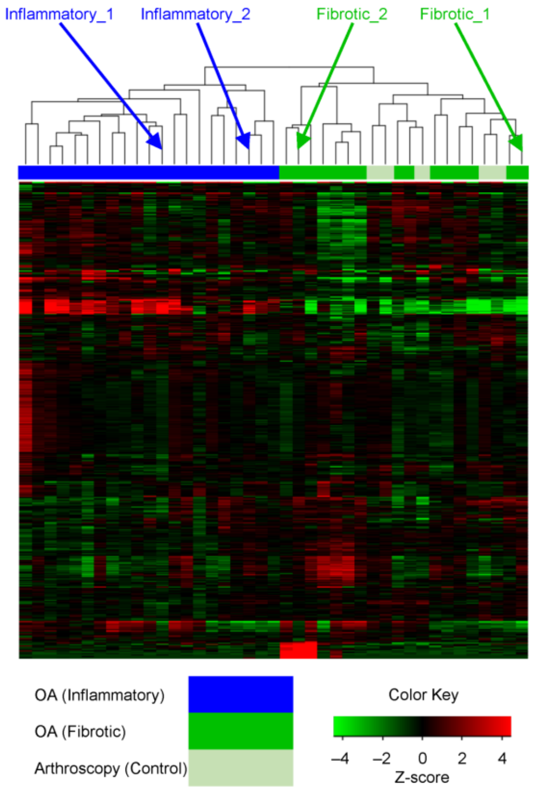

Supplemental Figure 3. Four samples of knee OA synovium submitted to scRNA-seq from the inflammatory and fibrotic types.

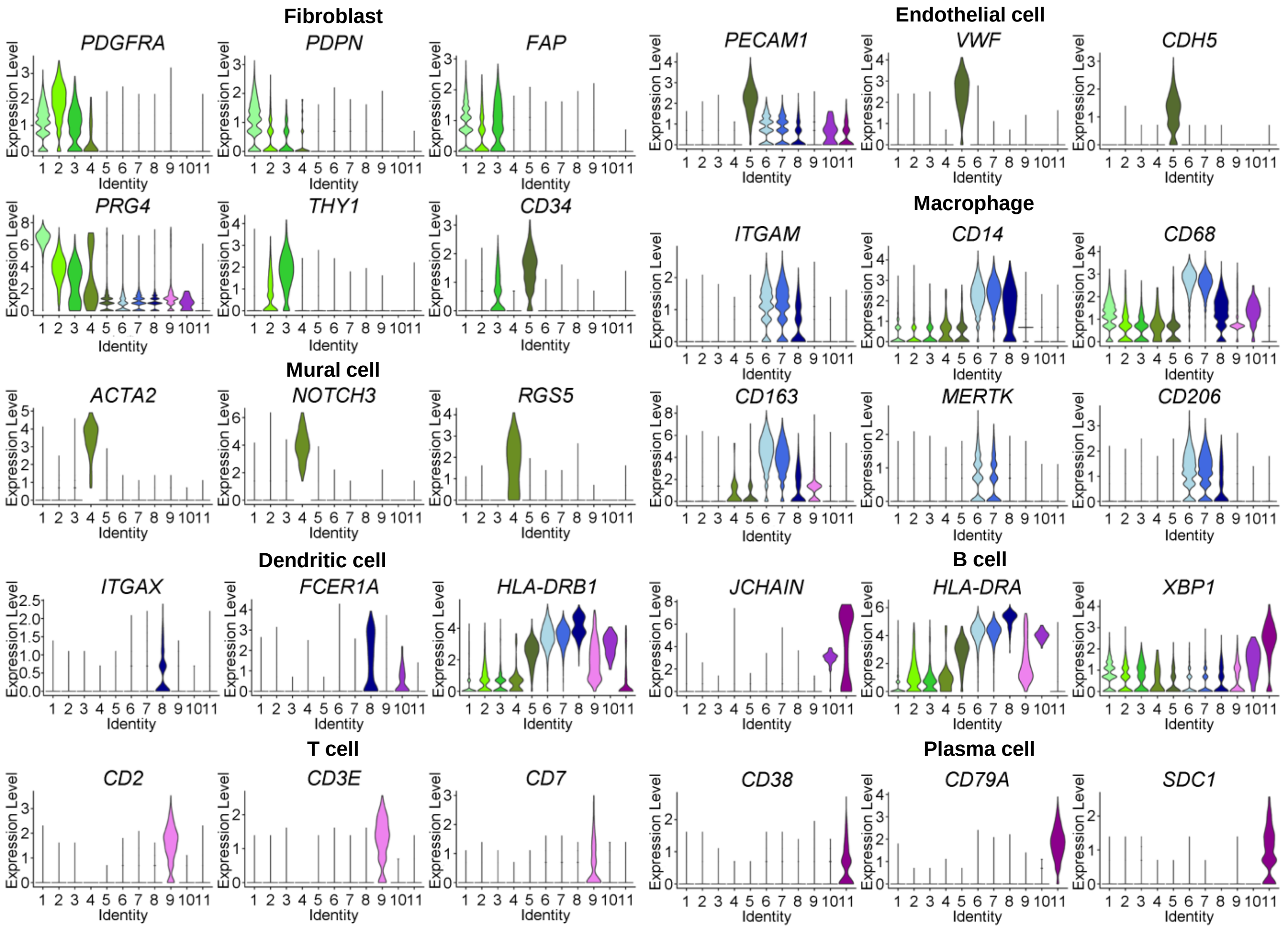

Supplemental Figure 4. Violin plots of marker genes for synovial cell subsets.

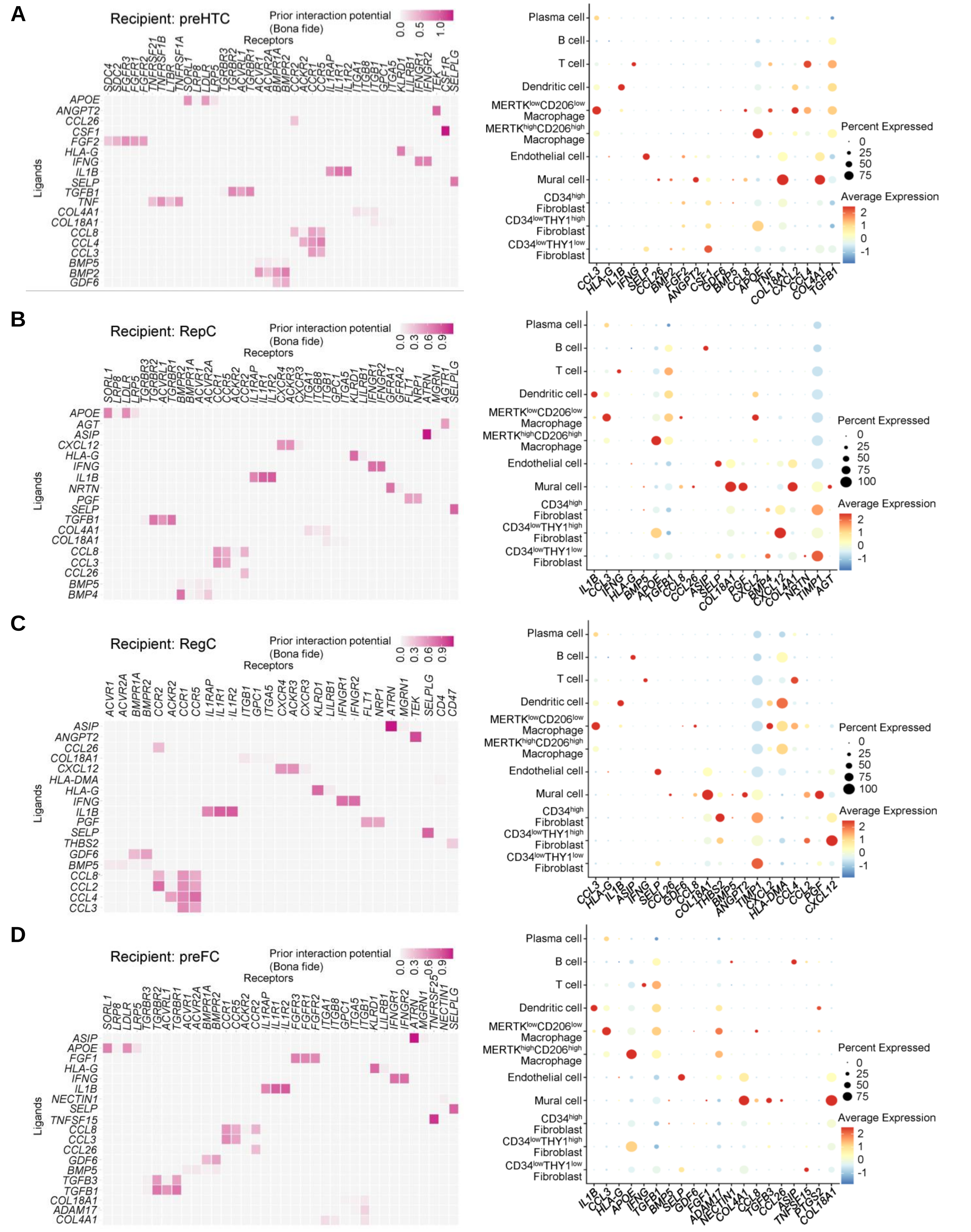

Supplemental Figure 5. Estimated interactions between synovial cell subsets and articular chondrocytes using the merged datasets. (A-D) Ligand-receptor analyses between each of three representative chondrocyte subsets and all synovial cell and chondrocyte subsets. Each of the three chondrocyte subsets, i.e., preHTC: Pre-hypertrophic chondrocyte (**A**), RepC: Reparative chondrocyte (**B**), RegC: Regulatory chondrocyte (**C**), and preFC: Pre-fibrotic chondrocyte (**D**), was set as the recipient. Left panels: heat maps of ligand-receptor interaction potential. Right panels: dot plots of the top 20 ligand gene expression profiles.

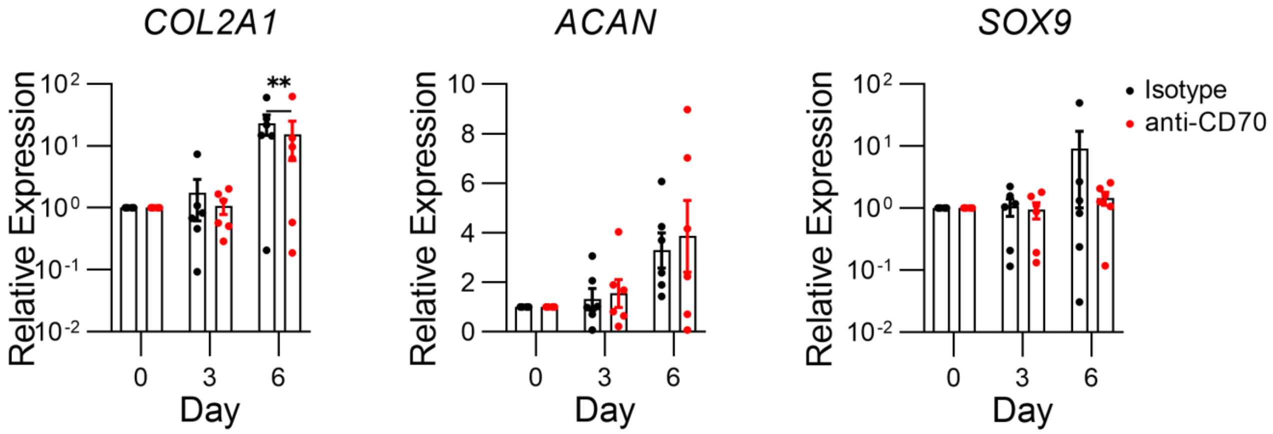

Supplemental Figure 6. Time-course mRNA levels in cultured cartilage tissues. Time-course mRNA levels of *COL2A1*, *ACAN*, and *SOX9* in cultured cartilage tissues with anti-CD70 antibody or isotype control. n = 6 biologically independent experiments. Data are expressed as dot plots and mean ± SEM. Mann-Whitney U test (*P < 0.05, **P < 0.01).

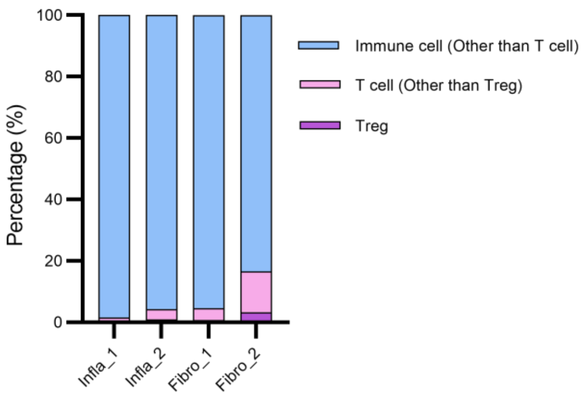

Supplemental Figure 7. Percentage of immune cell in the four synovium samples. Percentage of immune cells (other than T cells), T cells (other than Tregs) and Tregs in the four synovium samples. Infla: inflammatory group; Fibro: fibrotic group.

	Inflammatory (*n* = 21)	Fibrotic (*n* = 15)	*P*-value
**Age (yrs)**	71.2 ± 7.5	75.3 ± 9.1	0.15
**Sex (M/F)**	5/16	7/8	0.18
**BMI kg/m^2)**	26.6 ± 2.8	26.6 ± 3.8	0.55
**KL grade (3/4)**	12/9	8/7	0.82
**ROM (°)**	112.6 ± 17.7	117.8 ± 12.4	0.87

Supplemental Table 1. Demographic characteristics of the patients included in RNA-seq. The two groups of patients were categorized based on gene expression profiles. BMI: body mass index; KL: Kellgren-Lawrence; ROM: range of motion. Two-tailed Student's t test.

	Inflammatory (*n* = 21)		Fibrotic (*n* = 15)		
	Inflammatory_1 (*n* = 15)	Inflammatory_2 (*n* = 6)	Fibrotic_1 (*n* = 8)	Fibrotic_2 (*n* = 7)	*P*-value
**Age (yrs)**	70.4 ± 8.0	73.2 ± 6.1	73.5 ± 8.5	77.3 ± 10.0	0.44
**Sex (M/F)**	2/13	3/3	4/4	3/4	0.16
**BMI (kg/m^2)**	26.5 ± 2.9	27.2 ± 2.8	26.5 ± 4.4	27.0 ± 3.3	0.84
**KL grade (3/4)**	10/5	2/4	4/4	4/3	0.57
**ROM (°)**	109.7 ± 18.7	120.0 ± 13.8	119.6 ± 13.7	115.7 ± 11.3	0.43

Supplemental Table 2. Demographic characteristics of the patients included in RNA-seq. The four subgroups of patients were categorized based on gene expression profiles. BMI: body mass index; KL: Kellgren-Lawrence; ROM: range of motion.

	Inflammatory_1	Inflammatory_2	Fibrotic_1	Fibrotic_2
**Age (yrs)**	63	74	76	86
**Sex (M/F)**	M	M	M	M
**BMI (kg/m^2)**	28.7	28.2	25.6	33.3
**Comorbidities**	Hypertension<br>Hyperlipidemia	Hypertension<br>Hyperlipidemia<br>Atrial fibrillation<br>Type 2 diabetes mellitus<br>Pancreatic cyst	Hypertension	Hypertension<br>Hyperlipidemia
**KL grade**	4	3	3	4
**ROM (°)**	115	120	130	110
**KOOS total score**	57.1	51.8	49.4	75.6
**KOOS symptom score**	46.4	75.0	53.6	89.3
**KOOS pain score**	52.8	50.0	50.0	86.1
**KOOS ADL score**	75.0	58.8	61.8	85.3
**KOOS sprots score**	40.00	20.0	20.0	25.0
**KOOS QOL score**	31.3	25.0	25.0	50.0

Supplemental Table 3. Demographic characteristics of the four patients included in scRNA-seq. BMI: body mass index; KL: Kellgren-Lawrence; ROM: range of motion; KOOS: knee injury and osteoarthritis outcome score.

Supplemental Table 4. Antibodies and other reagents used for immunofluorescent staining.

	Supplier	Catalog #
Podoplanin Monoclonal Antibody (NZ-1.3)	eBioscience	14-9381-82
Anti-CD34 Antibody (EP373Y)	Abcam	ab81289
Human CD27 Ligand/TNFSF7 Antibody	R & D Systems	MAB2738
Goat anti-Rat IgG (H+L) Secondary Antibody, Alexa Fluor 488	Invitrogen	A-11006
Goat anti-Rabbit IgG (H+L) Secondary Antibody, Alexa Fluor 555	Invitrogen	A-32732
Alexa Fluor 647 Tyramide Reagent	Invitrogen	B-40958
TrueBlack Lipofuscin Autofluorescence Quencher, 20X in DMF	Biotium	58889-71
Fluoro-KEEPER Antifade Reagent, Non-Hardening Type with DAPI	Nacalai Tesque	12745-74

Supplemental Table 5. Antibodies and other reagents used for FCM.

	Supplier	Catalog #
CFSE Cell Division Tracker Kit	BioLegned	423801
Human TruStain FcX	BioLegned	422301
Brilliant Violet 421 anti-human CD194 (CCR4) antibody	BioLegned	359413
Brilliant Violet 510 anti-human CD45 antibody	BioLegned	304035
FITC anti-human CD3 antibody	BioLegned	300405
FITC anti-human IL-1β antibody	BioLegned	511705
PE/Dazzle 594 anti-human CD183 (CXCR3) antibody	BioLegned	353735
PE/Dazzle 594 anti-human CD3 antibody	BioLegned	300449
PerCP/Cyanine 5.5 anti-human CD70 antibody	BioLegned	355107
PE anti-human CD25 antibody	BioLegned	302605
PE anti-human CD8 antibody	BioLegned	344705
PE/Cyanine 7 anti-human CD127 (IL7Rα) antibody	BioLegned	351320
PE/Cyanine 7 anti-human CD14 antibody	BioLegned	325617
APC anti-human CD4 antibody	BioLegned	317416
APC/Fire 750 anti-human CD27 antibody	BioLegned	356427
Monensin Solution (1,000X)	BioLegned	420701
IC Fixation Buffer	Invitrogen	00-8222
Permeabilization Buffer 10X	Invitrogen	00-8333

Supplemental Table 6. Primers used for RT-qPCR.

Gene Symbol		Sequence
GAPDH	F	ACCCAGAAGACTGTGGATGG
	R	TTCTAGACGGCAGGTCAGGT
IL1B	F	ACGATGCACCTGTACGATCA
	R	TCTTTCAACACGCAGGACAG
IL6	F	AGACAGCCACTCACCTCTTCA
	R	AGTGCCTCTTTGCTGCTTTC
TNF	F	CAGAGGGCCTGTACCTCATC
	R	GGAAGACCCCTCCCAGATAG
COL2A1	F	GGTGGCTTCCATTTCAGCTA
	R	TACCGGTATGTTTCGTGCAG
ACAN	F	ACAGCTGGGGACATTAGTGG
	R	GTGGAATGCAGAGGTGGTTT
SOX9	F	ATGCAAGCATGTGTCATCCA
	R	AGGTCTGTCAGTGGGCTGAT
